# Establishing a model fish for the Neotropical region: The case of the yellowtail tetra *Astyanax altiparanae* in advanced biotechnology

**DOI:** 10.3389/fgene.2022.903990

**Published:** 2022-11-30

**Authors:** George Shigueki Yasui, Nivaldo Ferreira do Nascimento, Matheus Pereira-Santos, Amanda Pereira dos Santos Silva, Geovanna Carla Zacheo Coelho, José Antônio Visintin, Fábio Porto-Foresti, Laura Satiko Okada Nakaghi, Norberto Castro Vianna, Gabriela Braga Carvalho, Paulo Sérgio Monzani, Lucia Suárez López, José Augusto Senhorini

**Affiliations:** ^1^ Laboratory of Fish Biotechnology, National Center for Research and Conservation of Continental Fish, Chico Mendes Institute of Biodiversity Conservation, Brasília, Brazil; ^2^ Department of Animal Reproduction, Faculty of Veterinary Medicine, University of São Paulo, São Paulo, Brazil; ^3^ Peixetec Biotecnologia Em Organismos Aquáticos LTDA, São Paulo, Brazil; ^4^ Graduate Course of Biological Sciences (Zoology), São Paulo State University, São Paulo, Brazil; ^5^ Academic Unit, Federal Rural University of Pernambuco (UFRPE), Serra Talhada, Brazil; ^6^ Federal Rural University of Rio de Janeiro, Animal Science Graduate Program, Seropédica, Brazil; ^7^ Department of Biological Sciences, São Paulo State University, São Paulo, Brazil; ^8^ Aquaculture Center, São Paulo State University, São Paulo, Brazil; ^9^ China Three Gorges Corporation (CTG), Beijing, China

**Keywords:** chromosome set manipulation, experimental fish, germline chimera, laboratory fish, micromanipulation, polyploids

## Abstract

The use of model organisms is important for basic and applied sciences. Several laboratory species of fishes are used to develop advanced technologies, such as the zebrafish (*Danio rerio*), the medaka (*Oryzias latipes*), and loach species (*Misgurnus* spp*.*). However, the application of these exotic species in the Neotropical region is limited due to differences in environmental conditions and phylogenetic distances. This situation emphasizes the establishment of a model organism specifically for the Neotropical region with the development of techniques that may be applicable to other Neotropical fish species. In this work, the previous research efforts are described in order to establish the yellowtail tetra *Astyanax altiparanae* as a model laboratory species for both laboratory and aquaculture purposes. Over the last decade, starting with artificial fertilization, the yellowtail tetra has become a laboratory organism for advanced biotechnology, such as germ cell transplantation, chromosome set manipulation, and other technologies, with applications in aquaculture and conservation of genetic resources. Nowadays, the yellowtail tetra is considered the most advanced fish with respect to fish biotechnology within the Neotropical region. The techniques developed for this species are being used in other related species, especially within the characins class.

## Introduction

Biological models are important to develop technologies in basic and applied sciences. In fish studies, the main application of model organisms focuses on small laboratory species and species for aquaculture production. Several fish species arose as laboratory species worldwide, such as the zebrafish (*Danio rerio*) (Westerfield, 2007; Feitsma and Cuppen, 2008; Dai et al., 2014), the medaka (*Oryzias latipes*) (Wittbrodt et al., 2002), and the loach (*Misgurnus* spp.) (Kostomarova, 1991; Arm, 2003), among other species (see Table 1). For the Neotropical region, there has been no model organism established for the laboratory work. A model organism specifically for the Neotropical region may improve the technologies in aquaculture, and the data are directly applicable for local conditions, as well as for other related species. Thus, the yellowtail tetra is considered a candidate for the model organism for the Neotropical region (Yasui et al., 2020c).

**TABLE 1:** Biological characteristics of some model fish species.

Species	Size	Sex maturation	Fecundity (egg/female)	Blood sampling	*In vitro* fertilization
Yellowtail tetra (*Astyanax altiparanae*)	4–15 cm ^(1)^	4 months ^(1)^	11,086–31,720 ^(2)^	Feasible ^(3)^	Easy
Loach (*Misgurnus anguillicaudatus*)	13–17 cm ^(4)^	1–2 years ^(5)^	1,800–15,500 ^(6)^	Feasible ^(7)^	Easy
Medaka (*Oryzias latipes*)	3–4 cm ^(8)^	2 months ^(9)^	30–50/day ^(8)^	Feasible ^(10)^	Moderate
Stickleback (*Gasterosteus aculeatus*)	2.5–8 cm ^(11)^ < 10 cm ^(12)^	1–2 years ^(13)^	161–4,130 ^(14)^	Feasible ^(15)^	Difficult
Zebrafish (*Danio rerio*)	2.5–4.5 cm ^(16)^	2.5–3 months ^(17)^	300/week ^(18)^	Feasible ^(19)^	Moderate

1: Yasui et al. (2020c); 2: Sato et al. (2006); 3: Nascimento et al. (2020b); 4: Gao et al. (2014); 5: Lei and Sinica, (2020); 6: Suzuki (1983); 7: Gao et al. (2007); 8: Wittbrodt et al. (2002); 9: Wakamatsu et al. (2001); 10: Niimi and Imada, (2008); 11: Olsson et al. (2019); 12: Cresko et al. (2007); 13: Mehlis and Bakker, (2013); 14: Patimar et al. (2010); 15: Wirzinger et al. (2007); 16: Clark et al. (2018); 17: Nasiadka and Clark, (2012); 18: Hsu et al. (2007); 19: Carradice and Lieschke, (2008).

Several biological characteristics make this species a prime candidate for laboratory studies, including: 1) small size; 2) domestication into artificial conditions (aquaria and dry food); 3) early sex maturation (4–5 months); 4) easy breeding management for *in vitro* fertilization; and 5) external sexual dimorphism (Arai, 2001; Wittbrodt et al., 2002; Westerfield, 2007; Yasui et al., 2020c).

Although the use of laboratory species is interesting and may accelerate several technologies in the field of genetics and biotechnology, it is necessary to first establish the basic information for the successful application of a laboratory fish. Determination of characteristics, such as feeding and maintenance in the laboratory, environmental conditions (photoperiod, aquarium size, temperature for maintenance, and reproduction, etc.), reproduction, larvae culture, and disease treatment and prevention, is first necessary to then advance into other techniques such as transgenesis and chromosome manipulation. Most of the research topics in the field of biotechnology, such as transgenesis (Stahl et al., 2019), chromosome set manipulation (Dunham, 2004), primordial germ cell (PGC) transplantation (Yamaha et al., 2010), intracytoplasmic sperm injection (ICSI) (Yasui et al., 2018), and other biotechnological approaches, require the manipulation of embryos during the early stages, and therefore, knowledge of fertilization timing is necessary.

This review shows the main techniques developed in the field of biotechnology to establish the yellowtail tetra as the most advanced laboratory fish native to the Neotropical region.

## The yellowtail tetra *Astyanax altiparanae*

The generic name “tetra” denotes several small-bodied species of fishes belonging to Characidae from the Neotropical region, although an African group also exists (subfamily Alestidae). The name “tetra” was originated from the genus *Tetragonopterus*, an important genus in this group. In this group, the genus *Astyanax* is widely distributed across America, from the south of Argentina to North America. Although tetra species are commonly associated with aquarium fish trade, some aquaculture species intended for production also exist, such as the yellowtail tetra *Astyanax altiparanae* (Garutti and Britski, 2000). This fish is a small-bodied species (12–15 cm) and largely distributed throughout the Neotropical region. *Astyanax altiparanae* is considered to be a junior synonym of *Astyanax lacustris* by Lucena and Soares, (2016). However, this recent classification is still not unanimously agreed upon by ichthyologists. Therefore, in the present review, the traditional classification will be used, and the name *Astyanax altiparanae* (Garutti and Britski, 2000) will be adopted.

**FIGURE 1:**
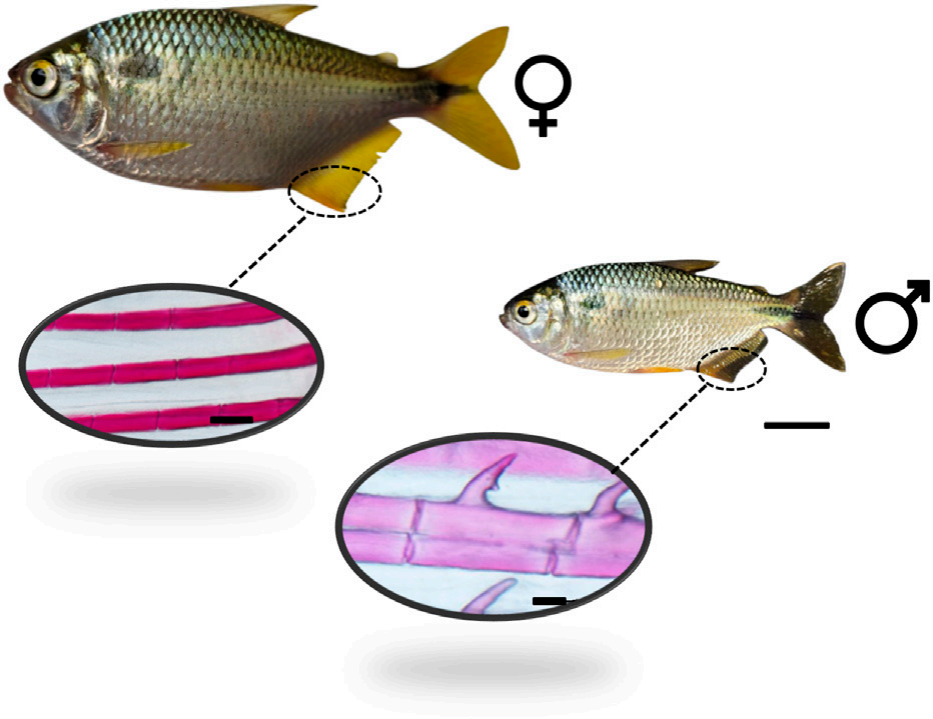
Adult male and female yellowtail tetra *Astyanax altiparanae*. The male presents bony hooks in the anal fin, which are not present in the females. Bar scale is 1 cm for the fish, 50 µm for the male fin, and 100 µm for the female fin.

The yellowtail tetra adapts easily into aquaria, aquaculture tanks, and ponds, and it can be fed with artificial commercial pellets. The intertidal spawning pattern allows it to be bred year-round, given that the temperature and photoperiod are manipulated (Machado-Evangelista et al., 2019). The yellowtail tetra presents sexual dimorphism as shown in our recent study (Siqueira-Silva et al., 2020), noting that the male presents bony hooks in the anal and ventral fins that are not present in the females (Figure 1).

The yellowtail tetra is an interesting model species to develop biotechniques because experiments may be conducted in aquaria year-round. In addition, the results are applicable to other species, especially other characin species that include more than 1,150 species (Nelson et al., 2016).

## The first step in fish biotechnology: Sperm storage and *in vitro* fertilization

For biotechnological studies, such as chromosome set manipulation and germ cell transplantation, it is necessary to manipulate the fertilization timing. In the case of the yellowtail tetra, studies regarding reproduction were previously conducted using natural spawning (without hormonal treatment) and semi-natural spawning (hormonal treatment followed by spontaneous spawning) (Garutti, 1989, 2003; Veloso-Júnior et al., 2009; Weber et al., 2013). Artificial insemination was conducted in *A. bimaculatus* (Sato et al., 2006); however, the spermatozoa were not immobilized, and then, the timing of gamete activation was not controlled.

The first step to establish the yellowtail tetra in genetic and reproductive studies was to collect gametes and succeed with *in vitro* fertilization. Based on the loach *Misgurnus anguillicaudatus* procedures (Yasui et al., 2010, 2011), Yasui et al. (2015) evaluated several hormonal treatments for gamete maturation and then established sperm sampling and refrigerated storage in an extender. The extender allows one to immobilize and control the timing of sperm activation and fertilization. Oocyte sampling using Petri dishes was also important for laboratorial management for subsequent gamete and embryo manipulation. The same authors showed that oocyte storage was not possible in the species. After the studies mentioned previously, other advances in the reproduction of the yellowtail tetra have also been published (Brambila-Souza et al., 2021; Roza de Abreu et al., 2021).

## Knowledge of gamete characteristics

**FIGURE 2:**
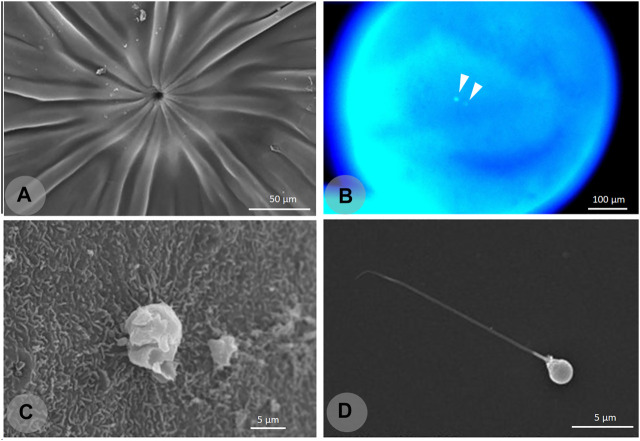
Cytological and ultrastructural images from the oocytes and spermatozoa of the yellowtail tetra *Astyanax altiparanae*. Ultrastructural analysis (scanning electron microscopy,SEM) of the oocyte micropyle **(A)** showing grooves that guide the sperm entry. DAPI staining of the fertilized oocyte showing male and female pronucleus **(B)**. Second polar body extrusion **(C)**. Spermatozoon of the yellowtail tetra **(D)**. This information was important to develop chromosome set manipulation techniques.

After artificial propagation (Yasui et al., 2015), the next step in this line of research was to investigate basic characteristics of the gametes and embryo development. Pereira-Santos et al. (2016) analyzed the gametes, including ultrastructural analysis, second polar body extrusion, pronucleus fusion, and embryonic development at different temperatures. The spermatozoa of *A. altiparanae* have a typical morphology of the teleost fish, presenting a spherical head (1.88 µm), a midpiece (0.75 μm), and a single flagellum (18.67 μm). Temperature significantly influenced the development, where hatching occurred at 25 h post-fertilization (hpf) at 22°C, 16 hpf at 26°C, and 11 hpf at 30°C. At 22°C, extrusion of the second polar body occurred at 6 min post-fertilization (mpf), (Figure 2) and pronucleus fusion occurred at 10 mpf. This basic information gave important support for later works with chromosome manipulation and germ cell transplantation.

The first attempt to use computer-assisted sperm analysis (CASA) (Sperm Class Analyzer, Microptic, Barcelona, Spain) evaluated motility (%), linearity (LIN), beat cross-frequency (BCF), amplitude of lateral head (ALH), curvilinear velocity (VCL), straight line velocity (VSL), average path velocity (VAP), wobble (WOB), and straightness (STR). At 15 and 45 s post-activation, motility percentages were 83.9 ± 3.1% and 54.5 ± 5.5%, respectively, and the mean motility duration was 75 s. Sperm were maintained at 2.5°C in modified Ringer’s solution (128.3 mM NaCl, 23.6 mM KCl, 3.6 mM CaCl_2_, and 2.1 mM MgCl_2_) with good viability up to 3 days and later. Other studies implemented CASA with open-source software and successfully used it in experimentation on yellowtail tetra with similar results, using simpler parameters such as total motility, curvilinear velocity (VCL), average path velocity (VAP), and straight line velocity (VSL) (Gonçalves et al., 2018; Rocha et al., 2020).

The study of oocytes and fertilization success rates was also conducted (Pereira-Santos et al., 2017), and the researchers concluded that *A. altiparanae* has one of the lowest insemination doses among teleosts (2,390 spermatozoa. oocyte^−1^ ml^−1^). Those conclusions were attributed to the small oocyte diameter (695.119 µm), large micropyle (7.57 µm), long motility duration (>75 s), and also the grooves in the oocytes surface that can guide the spermatozoa into the micropyle to optimize fertilization efficacy. This set of information was an important database to initiate advanced studies described in the following sections. The information obtained in the aforementioned studies also opened up new possibilities for approaches such as dispermic fertilization, since the micropyle diameter is greater than that of two sperm heads.

## Chromosome set manipulation

Chromosome manipulation in fish refers basically to polyploidy (triploids and tetraploids, etc.) and uniparental inheritance induced by gynogenesis and androgenesis. Artificially induced polyploids focus on the production of triploids and tetraploids for large-scale production of sterile fish. Inhibition of second polar body extrusion, achieved by heat, cold, pressure, or chemical treatments, gives rise to triploid progenies (Dunham, 2004). The inhibition of second mitotic division by similar treatments may induce tetraploids (Zhang and Onozato, 2004). The main procedures for chromosome manipulation in the yellowtail tetra are listed in the following sections.

### Triploids and hybrid triploids: Searching for sterile fish

Sterile yellowtail tetra is important for aquaculture because sterile fish present increased growth performance. In the field of conservation, sterile fish are important to avoid negative environmental impact from escaping, since in the Neotropical region, the introduction of exotic species is the second major cause of species endangerment (ICMBio, 2018). In addition, sterile fish may be a good recipient for cells of endangered species for subsequent surrogate propagation (Yamaha et al., 2001, 2007; Takeuchi et al., 2003), later serving as a repository gene bank. Previous attempts to obtain sterile yellowtail tetra were conducted by the depletion of germ cells, but the approach did not succeed a hundred percent in producing all sterile fish (Siqueira-Silva et al., 2015), so our group focused on polyploidization.

Initially, basic cytology information for chromosome set manipulation, including timing for second polar body release and fusion of male and female pronucleus, was studied (Santos et al., 2016). Based on such information, a more precise timing for diploidization and second polar body retention was established. This generated high percentages of triploids produced by using heat shock (40°C for 2 min) at 2 mpf, which guarantees 97.44% of triploids at the larvae stage (Adamov et al., 2017). The growth and reproductive performance of triploids were then studied (Nascimento et al., 2017a), showing that triploid females are sterile (Nascimento et al., 2017a) and present an increased carcass yield (%) (Nascimento et al., 2017b) when compared with diploids. On the other hand, triploid males were not sterile (Nascimento et al., 2017a; 2017b), limiting their application in aquaculture. In a later study, 100% sterile fish were achieved using triploid hybrids (Piva et al., 2018) in a special crossing of *A. altiparanae* and *A. fasciatus.* In this set of experiments, oocytes from yellowtail tetra *Astyanax altiparanae* were inseminated with sperm from five males (*A. altiparanae*, *A. fasciatus*, *A. schubarti*, *Hyphessobrycon anisitsi*, and *Oligosarcus pintoi*) in order to produce several interspecies hybrids and triploid hybrids. Surprisingly, only one cross (*A. altiparanae* x *A. fasciatus*) generated sterile offspring, and the progenies did not present germ lineage in the gonads.

### The rise of spontaneously occurring triploids

Surprisingly, spontaneously occurring triploids arose in some progenies, even without any treatment for second polar body retention. This phenomenon led our group to investigate the rise of these triploids, and aged oocytes stored *in vivo* (Nascimento et al., 2018) and *in vitro* (Pereira-Santos et al., 2018) gave rise to these triploids, indicating that oocytes must be fertilized just after ovulation in order to prevent the rise of triploids.

### Tetraploids

Based on the timing of pronucleus fusion (within 10 min) studied by Pereira-Santos et al. (2016) and the temperature (40°C) for heat shock obtained by Adamov et al. (2017), a more precise tetraploidization procedure was established, improving the success rate of tetraploidization (Nascimento et al., 2020b). The procedures were optimized using temperature shock at 26 mpf (40°C for 2 min), followed by incubation at 26°C, and this resulted in 94.55% tetraploids at the larvae stage. This was the first reported tetraploids within the characin group. In addition, these protocols were recently improved (Martins et al., 2021), where it was observed that post-shock temperature (22°C, 26°C, and 28°C) affects tetraploid production in *A. altiparanae* and must be considered in future protocols. Tetraploid males and females were also able to produce viable and diploid spermatozoa and oocytes, respectively, which are capable of mass production (100%) of triploid fish (Nascimento et al., 2020b; Alves et al., 2022). As tetraploidization is difficult to achieve and viable lines are limited to a few species (Piferrer et al., 2009), the current protocol makes the yellowtail tetra one of the most successful species with respect to tetraploidization. The tetraploids are fertile, and they are being used to produce 100% triploid progenies.

### Gynogenesis: Searching for monosex female progenies

Female yellowtail tetra fish are large and present increased growth performance when compared to males (Nascimento et al., 2017b), emphasizing the need for the establishment of a monosex female population in aquaculture. Studies on sex chromosomes using induced gynogenesis were then conducted (Nascimento et al., 2020a), indicating that the species presents an XX sex-determining system. This was the first attempt in gynogenesis within a Neotropical species, and most of the progenies reached 100% females (three out of four crosses). The resultant males derived from gynogenetic progenies were studied, concluding they were functional males (Lázaro et al., 2021).

### Development of flow cytometric procedures

In order to assess the success of chromosome manipulation, flow cytometry is a valuable tool to confirm the ploidy status of the polyploids and the efficacy of chromosome inactivation and doubling in uniparental progenies (i.e., gynogenesis and androgenesis). An important step for chromosome manipulation in yellowtail tetra was to develop flow cytometric analysis using dorsal fin samples (Xavier et al., 2017). This technique is based on a two-step procedure with cell lysing and nuclear staining for the subsequent analysis. In addition, cold storage of the fin samples for the subsequent flow cytometric analysis was developed (Yasui et al., 2020a). Regarding chromosome manipulation in a Neotropical species, the yellowtail tetra was the first to be analyzed using flow cytometry.

## Larvae feeding under laboratory conditions

In aquaculture, the general procedure for larvae feeding and raising them into a juvenile stage consists of releasing the larvae into a fertilized pond containing plankton. On the other hand, it is important to feed the fish under laboratory conditions in small containers (Petri dishes, aquaria, or plastic containers). It was necessary then to understand in detail the adequate food for the yellowtail tetra. In order to fill this gap, Bertolini et al. (2018) evaluated different diets and concluded that artemia and dry food optimized the growth and survival under laboratory conditions, giving them a routine for dependable growth of larvae into the juvenile stage.

## Prevention of sperm activation by urine

In small-bodied fish like the yellowtail tetra, sperm sampling is difficult to achieve because of small size. In the yellowtail tetra, the sperm is collected using a 1,000-µL micropipette. However, urine and sperm are released during sperm sampling, and the urine activates sperm motility and decreases the fertilization ability. The problem was partially solved using an immobilizing solution to re-immobilize the sperm (Yasui et al., 2015), although some activation still occurs. In a recent study (Rocha et al., 2020), the problem of activation was solved by maintaining males in a hyperosmotic environment (1% NaCl) for a few hours. The males were induced to spermatization and maintained in 1% NaCl for 6 h. The hyperosmotic environment concentrates the urine and does not activate the sperm motility at sampling. This procedure is now used for sperm sampling under laboratory conditions.

## Surrogate propagation using yellowtail tetra

Surrogate propagation denotes a fish producing gametes from other fish. This approach is interesting for aquaculture and conservation of genetic resources. Considering the yellowtail tetra as a model organism, this species may be used to produce gametes from endangered fish species. In addition, cells from endangered species may be cryopreserved in liquid nitrogen, serving later as a repository gene bank. The yellowtail tetra is being used as a model for other endangered characin species, such as *Brycon orbignyanus*. Other aquaculture characins, such as the streaked prochilod (*Prochilodus lineatus*) and pacu (*Piaractus mesopotamicus*) (Coelho et al., 2019, 2021, respectively), are also being studied to serve as cell donors, using yellowtail tetra to produce gametes from those species. As the sterile host was already established (Piva et al., 2018), transplantation procedures are now being established for several characin species.

**FIGURE 3:**
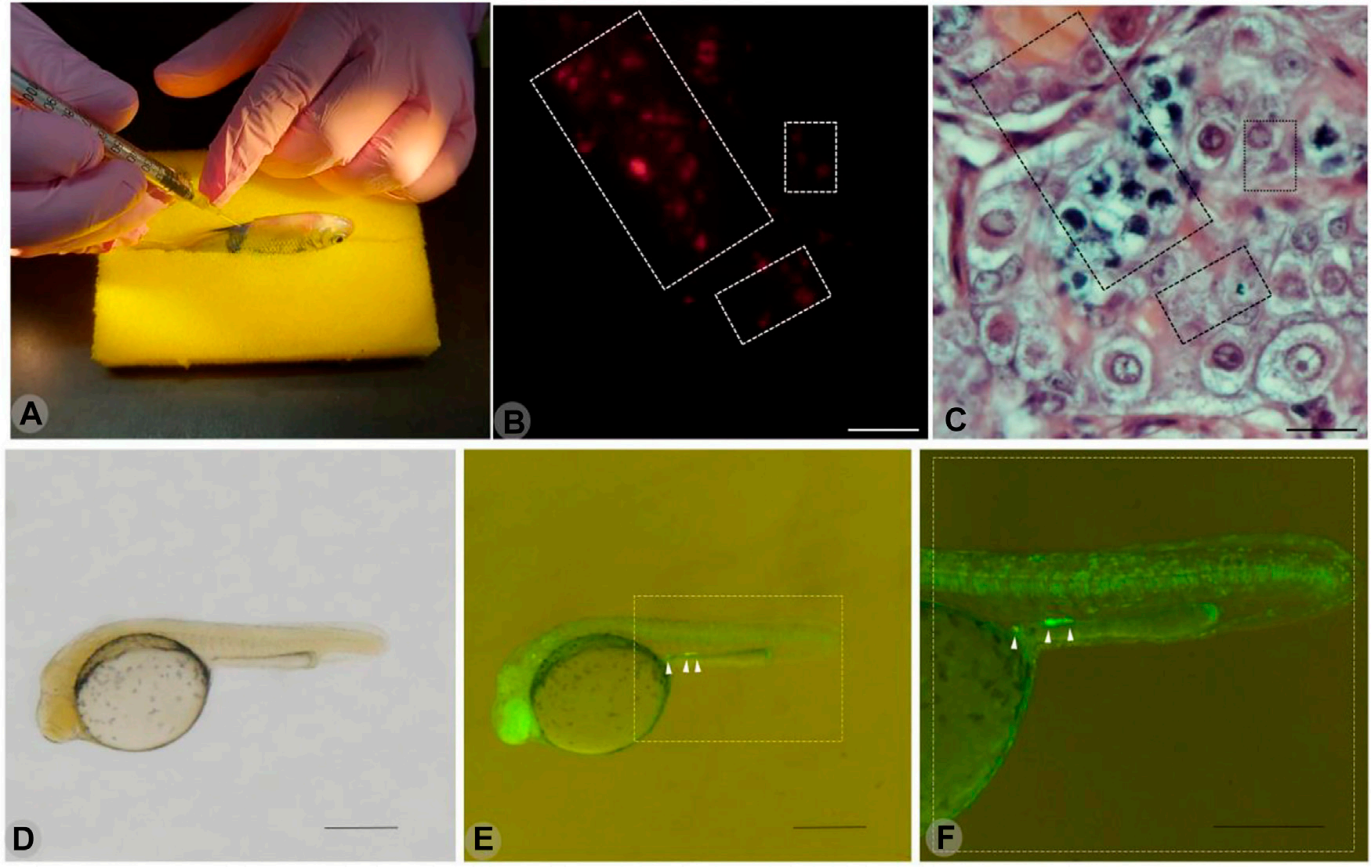
Transplantation of spermatogonial stem cells from endangered *Brycon orbignyanus* through the papillae of sterile juvenile yellowtail tetra *Astyanax altiparanae* **(A)**. Detached area indicates the testis containing transplanted cells stained with PKH26 and observed under fluorescence microscopy **(B)**. Same histological section visualized under normal light, and hematoxylin-eosin staining shows spermatocytes and spermatogonia from *B. orbignyanus*. Scale **(B–C)**: 50 µm. Below is a yellowtail tetra embryo injected with GFP-nos1 3′UTR mRNA from *Danio rerio*, observed under normal light **(D)**.Same embryo, observed under fluorescence microscopy, shows the presumptive primordial germ cells (PGCs) with GFP expression **(E)**. Gonadal ridge area in detail **(F)**.

The PGCs of the yellowtail tetra were identified *in vivo* using a GFP-nos1 3′UTR mRNA from *Danio rerio*. The injection of this artificial mRNA resulted in the expression of GFP in the PGCs (Figure 3). In addition, the spermatogonial stem cells from endangered *B. orbygnianus* were successfully transplanted into sterile adults of yellowtail tetra (Figure 3). Those procedures are being established as repository procedures for endangered species.

## The use of yellowtail tetra in other studies

The yellowtail tetra is also being used in other studies. For instance, based on the first attempt to analyze sperm motility by CASA (Pereira-Santos et al., 2016), motility parameters are being used in toxicological studies about pollutants, such as herbicides (Gonçalves et al., 2018) and aluminum (Pinheiro et al., 2020). The embryos and adults of yellowtail tetra species were used to evaluate toxicity of cyanopeptides (Fernandes et al., 2019) and aflatoxins (Michelin et al., 2017). An immunological study using triploids was conducted by Levy-Pereira et al. (2021), who observed that the cell counts of erythrocytes, leukocytes, and neutrophils were lower in triploid fish than diploids. Triploid erythrocytes were also larger with higher frequencies of abnormalities. Differences in gene expression related to immune response were also observed, reporting the lower expression of cytokine IL-1 (in the head, kidney, liver, and spleen) and TGF-β (in the spleen) in triploids. These results indicate that triploid fish present impaired immune systems and probably lower resistance to diseases. However, future studies involving pathogen challenges are necessary to confirm these assumptions.

## Application of technologies in other species

Based on the results obtained for yellowtail tetra, several advances within other Neotropical species were established. Using the same temperature (40°C) to induce triploids in the yellowtail tetra, triploid progenies were obtained for the streaked prochilod *Prochilodus lineatus* (Yasui et al., 2020b) and *Brycon amazonicus* (Nascimento et al., 2021). With some minor modifications, triploids and tetraploids were also generated when the temperature was set at 38°C for *Pimelodus maculatus* (Bertolini et al., 2020) and *Rhamdia quelen* (Garcia et al., 2017; García et al., 2017). *B. amazonicus* (Da Silva et al., 2017), *Pimelodus maculatus* and *Pseudopimelodus mangurus* (Arashiro et al., 2018), and *P. lineatus* (Coelho et al., 2019) were also studied in the field of embryology using the same procedures and temperatures (22°C, 26°C, and 30°C) as used for the yellowtail tetra. The same protocol for cytometric analysis established in the yellowtail tetra (Xavier et al., 2017) was used to identify polyploids in *B. amazonicus* (Nascimento et al., 2021), *P. lineatus* (Yasui et al., 2020b), *P. maculatus* (Bertolini et al., 2020), and *R. quelen* (Garcia et al., 2017).

## Future directions

Development of molecular markers is a priority for the yellowtail tetra, since it is the main confirmation tool for paternity within androgenesis, gynogenesis, and surrogate propagation. Techniques regarding cryopreservation of unusual fish developed recently are also being developed, such as gynogenetic, androgenetic, and polyploid genebanking. Cryopreservation of X spermatozoa obtained from spontaneously occurring gynogenetic males will be important to obtain monosex female populations. Regarding aquaculture, the establishment of sterile female populations will increase growth performance and also avoid the negative environmental impact caused by fish escaping. Transgenesis, single and multiple ICSI, and germ cell transplantation are among the ongoing studies in our group. However, several biotechniques currently used in other fish species have potential for *A. altiparanae*, such as the use of single nucleotide polymorphisms (SNPs), CRISPR/Cas9, transgenic, and microRNAs (miRNAs), and are discussed in the following paragraphs.

Clustered regularly interspaced palindromic repeats (CRISPR/Cas9) are DNA sequences that are used in genome editing technology. This technology, unprecedented in native fish species, has been increasingly used in aquaculture to manipulate reproduction and omega-3 content, growth, and metabolism (Straume et al., 2020; Sun et al., 2020), and the main model species for CRISPR/Cas9 in fish is the zebrafish (*Danio rerio*) (Chaudhary et al., 2020).

Transgenic fish, on the other hand, have already been developed for many fish species, such as the medaka (*Oryzias latipes*), zebrafish (*Danio rerio*), rainbow trout (*Oncorhynchus mykiss*), and loach (*M. anguillicaudatus*). Transgenic organisms receive DNA sequencing by artificial methods, incorporating one or more sequences into their chromosomal DNA. This type of animal is generally produced by microinjection or electroporation of newly fertilized oocytes or unfertilized gametes (egg or spermatozoa) (Maclean and Laight, 2000; Chen and Chen, 2020). Among the potential applications of transgenics in aquatic organisms, the increase in growth (Nam et al., 2001), tolerance to temperature (Cortemeglia and Beitinger, 2005) and salinity (El-Zaeem et al., 2014; Bystriansky et al., 2017), resistance to diseases (Dunham, 2009), and induction of sterility (Yu et al., 2011) are highlighted.

The SNPs are produced by mutations that occur in the genome, and its analysis has offered several applications in fish biology and aquaculture. An SNP marker was developed by liver transcriptome sequencing in pacu (*Piaractus mesopotamicus*), enabling a broad understanding of the population structure in the species and the possibility of elucidating adaptive mechanisms and manipulation of assisted reproduction. The establishment of SNPs also enabled the selection of groups with better genetic variability for storage and production (Mastrochirico-Filho et al., 2016).

The use of miRNAs, which are small and non-protein-coding RNA sequences, has increased the knowledge about several aspects of biological regulation mechanisms in animals and plants (Bizuayehu and Babiak, 2014; Mennigen, 2016). In fish, miRNAs are involved in several biological functions, such as regulation of development, organogenesis, growth, immune response, and reproduction (Mennigen, 2016; Andreassen and Høyheim, 2017; Tang et al., 2019; Zayed et al., 2019). As the study with miRNAs in fish are relatively recent and focused on a few species, more efforts are necessary.

In light of this, *A. altiparanae* can be considered the perfect model for Neotropical fish mainly because it is used in both basic and applied studies, such as aquaculture.

## Discussion

The establishment of a model fish is strategic for basic and applied sciences. The yellowtail tetra is being successfully used for this purpose. Triploids, tetraploids, gynogenetic, and chimeric fish were successfully developed by means of advanced biotechnologies (Adamov et al., 2017; Nascimento et al., 2020a, 2020b). In the Neotropical region, the yellowtail tetra became the most advanced with regards to such technologies, having important implications in embryology, genetics, reproduction, cryobiology, and even in medical sciences such as flow cytometry (Santos et al., 2016; Xavier et al., 2017; Yasui et al., 2020a). Several characin species present critical reproduction challenges or are considered endangered (Silveira and Straube, 2008). Some of the migratory aquaculture species such as *Prochilodus lineatus*, *Piaractus mesopotamicus*, *Colossoma macropomum*, *Brycon amazonicus*, and *Salminus brasiliensis* present large size (2–15 kg), and sex maturation occurs within 2–3 years (Hainfellner et al., 2012; Pardo-Carrasco et al., 2006; da Costa and Mateus, 2009; Almeida et al., 2016; Barzotto and Mateus, 2017). The yellowtail tetra, notably also a characin species, may then be used to produce gametes from these characins. This would facilitate the reproductive management and accelerate techniques for genetic improvement, which requires several successive generations (Arai, 2001; Du et al., 2021).

Despite their importance to aquaculture and inland fisheries, the development of biotechniques is difficult to achieve because spawning management is more difficult. However, some techniques developed for the yellowtail tetra may be promptly used in other species. For instance, triploidization of *Brycon amazonicus* (Nascimento et al., 2021) and *Prochilodus lineatus* (Yasui et al., 2020b) was successful when using the same procedures of heat shocking for second polar body retention (2 mpf for 2 min, 40°C). In addition, confirmation of the ploidy status using flow cytometry was achieved using the protocol established for yellowtail tetra (Xavier et al., 2017; Yasui et al., 2020a).

In the case of chromosome set manipulation, cytological observation of post-insemination events (second polar body extrusion and pronucleus fusion) gave a precise timing for the successful manipulation of the reproduction cycle. The observation of second polar body release by histological sections and scanning electron microscopy improved the production of triploids and gynogenetic progenies. The use of fluorescent dyes such as DAPI (4′,6-diamidino-2-phenylindole) to observe pronucleus fusion (Itono et al., 2006) gave a precise timing for diploidization. Most of the previous protocols to obtain triploids and tetraploids used trial-and-error procedures (Piferrer et al., 2009) without cytological observations, which is time-consuming and more difficult to achieve.

Toxicological studies conducted on the yellowtail tetra are being used to establish safe concentrations for the environment (Gonçalves et al., 2018; Fernandes et al., 2019) and food products (Michelin et al., 2017), which also emphasize the importance of a model species.

Establishment of a model organism requires long-term research efforts for not only in the field of genetics and reproduction, but it also lays the foundation for complementary studies in the field of nutrition, physiology, animal behavior, water quality, immunology, pathology, and many other research fields. In addition, several confirmation tools become necessary for each of them (flow cytometry and water analysis, etc). In order to develop advanced biotechnologies in the yellowtail tetra, several initial stages were developed using trial-and-error experiments with most resulting in unpublished data, focusing on parameters that include container volume to maintain fish, materials (plastic and glass, etc.), type of food, substrate (as plants or tubes to avoid aggressive behavior), and many other steps that are time-consuming. Although it is simple to evaluate, in general, these kinds of bottlenecks are not published in the literature, and the successful procedures and parameters take long periods to be developed. As seen previously, establishing a model organism is thus a multidisciplinary task, for which teamwork is extremely necessary. In the case of the yellowtail tetra, a part of the laboratorial procedures were adapted and transferred from the loach *Misgurnus anguillicaudatus*, but several biological differences did not allow for advancement in some research fields. In the case of gamete sampling, transfer of the procedures was successful, but the transplantation of embryonic cells was not possible in the yellowtail tetra because of the enzymes for chorion digestion and solutions to maintain the denuded embryos. For example, the hatching period from fertilization to hatching, depending on the temperature, takes 2–3 days in the loach (Fujimoto et al., 2006) and approximately 60 days in salmonids (Danner, 2008), but in the yellowtail tetra, hatching occurs in only 11 h (Santos et al., 2016). This difference makes PGC transplantation much more difficult in the yellowtail tetra, since transplantation is possible only within a few minutes. On the other hand, other biological features of the yellowtail tetra are interesting, such as the possibility of blood sampling and a subsequent serum analysis. Notably, this is difficult to achieve in some small-bodied fish like the medaka and the zebrafish.

In conclusion, the yellowtail tetra is an emerging experimental fish model for several research fields, especially with regards to genetics and reproduction. Basic and advanced studies were carried out during the last decade in order to establish this species as a model organism, and it is currently considered the most advanced model organism in techniques such as germ cell transplantation, micromanipulation, and chromosome set manipulation. Other research efforts are still ongoing.
